# Dietary Interventions and Testosterone Levels in Obese Men: A Systematic Review Comparing the Mediterranean Diet, Ketogenic Diet, and Intermittent Fasting

**DOI:** 10.3390/nu18152417

**Published:** 2026-07-24

**Authors:** Sandro La Vignera, Rosita A. Condorelli

**Affiliations:** Department of Clinical and Experimental Medicine, University of Catania, Via S. Sofia 78, 95123 Catania, Italy; rosita.condorelli@unict.it

**Keywords:** obesity, testosterone, hypogonadism, ketogenic diet, Mediterranean diet, intermittent fasting, male obesity secondary hypogonadism, VLCKD, androgens, dietary intervention

## Abstract

Background/Objectives: Male obesity secondary hypogonadism (MOSH) is a highly prevalent condition characterised by reduced testosterone levels in obese men, driven by increased aromatase activity, reduced SHBG, insulin resistance, and HPG axis suppression. Dietary interventions represent a cornerstone of non-pharmacological management; however, the comparative efficacy of different dietary patterns on testosterone restoration remains unclear. Methods: We conducted a systematic literature review following PRISMA 2020 guidelines. Comprehensive searches were performed in SciSpace, Google Scholar, and PubMed through June 2026. Eligible studies included human adult males with obesity (BMI ≥ 30 kg/m^2^) undergoing Mediterranean diet (MedDiet), ketogenic diet (KD/VLCKD), or intermittent fasting (IF) interventions, with quantitative assessment of testosterone or androgen status. Results: From 697 initial records, 500 unique papers were identified after deduplication. Following abstract screening (*n* = 442 excluded) and full-text assessment (*n* = 6 excluded), 52 studies were included in the final synthesis. VLCKD demonstrated the most consistent evidence for testosterone improvement, with RCTs reporting significant increases in total testosterone (+1.5 to +3.0 nmol/L). Intermittent fasting showed promising but more heterogeneous results. Evidence for Mediterranean diet was limited, focusing primarily on metabolic rather than hormonal outcomes. Weight loss emerged as a critical mediator across all dietary interventions (~3 nmol/L per 10 kg weight loss). Conclusions: Among dietary interventions for MOSH, VLCKD shows the strongest evidence for testosterone restoration in obese men, through rapid weight loss, improved insulin sensitivity, reduced inflammation, and potential direct HPG axis effects. Intermittent fasting represents a viable alternative. Mediterranean diet, while beneficial for cardiovascular health, lacks robust interventional evidence for testosterone improvement in this population. Systematic Review Registration: This review was not prospectively registered. PROSPERO registration is recommended for future updates.

## 1. Introduction

Male obesity secondary hypogonadism (MOSH), also termed functional hypogonadism, represents one of the most prevalent endocrine disorders among men with excess adiposity [1,2]. Epidemiological data consistently demonstrate an inverse relationship between body mass index (BMI) and serum testosterone concentrations, with obese men exhibiting total testosterone levels 25–30% lower than their lean counterparts [3,4]. The clinical consequences of this hormonal deficit extend beyond reproductive dysfunction and encompass metabolic dysregulation, cardiovascular risk, impaired quality of life, and psychological morbidity [5,6].

The pathophysiology of MOSH is multifactorial and bidirectional. Adipose tissue hypertrophy increases the expression and activity of aromatase (CYP19A1), the enzyme responsible for peripheral conversion of testosterone to oestradiol, thereby creating a negative feedback loop that suppresses gonadotropin-releasing hormone (GnRH) pulsatility and, consequently, luteinising hormone (LH) secretion [7,8]. Concurrently, obesity-associated hyperinsulinaemia and insulin resistance reduce hepatic synthesis of sex hormone-binding globulin (SHBG), further diminishing bioavailable testosterone fractions [9]. Systemic low-grade inflammation—characterised by elevated IL-6, TNF-α, and CRP—exerts direct inhibitory effects on Leydig cell steroidogenesis [10,11]. The ‘Gut Endotoxin Leading to a Decline IN Gonadal function’ (GELDING) theory has been proposed as an additional mechanistic pathway, suggesting that obesity-induced intestinal dysbiosis and lipopolysaccharide (LPS) translocation may compromise testicular function [12]; however, direct causal evidence in humans remains limited, and this mechanism warrants further prospective investigation before definitive conclusions can be drawn.

Given the reversible nature of MOSH—in contrast to organic hypogonadism—lifestyle interventions, particularly dietary modifications, represent the primary therapeutic strategy before considering testosterone replacement therapy (TRT) [13,14]. Weight loss of 5–10% of body weight has been associated with clinically meaningful testosterone increments, and the magnitude of hormonal recovery appears proportional to the degree of weight reduction [15,16]. However, the optimal dietary approach to maximise testosterone restoration in obese men remains undefined.

Three dietary strategies have received particular attention in this context: (1) the Mediterranean diet (MedDiet), characterised by high consumption of plant foods, olive oil, moderate fish and poultry intake, and low red meat consumption, with established cardioprotective and anti-inflammatory properties [17,18]; (2) the ketogenic diet (KD) and its clinical variant, the very-low-calorie ketogenic diet (VLCKD), defined by severe carbohydrate restriction (<50 g/day) leading to nutritional ketosis, with demonstrated efficacy in rapid weight loss and metabolic improvement [19,20]; and (3) intermittent fasting (IF), encompassing various time-restricted eating (TRE) and alternate-day fasting (ADF) protocols [21,22]. These three dietary strategies were selected for synthesis on the basis of the volume of available evidence, their contrasting metabolic mechanisms, their established clinical relevance in obesity management, and their growing adoption in clinical and research settings, enabling a meaningful comparative synthesis.

Despite a growing body of literature on each of these dietary patterns individually, no systematic review has directly compared their relative efficacy in improving testosterone levels specifically in obese men. The rising global prevalence of male obesity, the growing recognition of MOSH as a distinct and clinically relevant entity, and increasing interest in dietary alternatives to testosterone replacement therapy (TRT) collectively underline the importance and timeliness of such a synthesis. This systematic review aims to fill this evidence gap by synthesising available data, evaluating the hormonal and metabolic outcomes of MedDiet, KD/VLCKD, and IF interventions, and providing evidence-informed recommendations for clinical practice.

## 2. Materials and Methods

### 2.1. PRISMA 2020 Compliance Statement

This systematic review was conducted and reported in accordance with the Preferred Reporting Items for Systematic Reviews and Meta-Analyses (PRISMA) 2020 statement [23]. The completed PRISMA 2020 checklist is provided as Supplementary Material S6 (Table S6).

### 2.2. Protocol and Registration

This review was not prospectively registered. The authors acknowledge that prospective registration in PROSPERO (https://www.crd.york.ac.uk/prospero/, accessed on 20 June 2026) represents a fundamental requirement of methodological rigour for systematic reviews, and the absence of registration constitutes a significant methodological limitation of the present work. Retrospective registration was not pursued following completion of the review. The authors are committed to prospective registration in PROSPERO for any future updates of this review or for related systematic reviews conducted in this field.

### 2.3. Eligibility Criteria

Studies were eligible for inclusion if they met the following PICO criteria:

- **Population (P):** Adult males (age ≥ 18 years) with obesity (BMI ≥ 30 kg/m^2^) or overweight with metabolic complications.
- **Intervention (I):** Mediterranean diet, ketogenic diet (including VLCKD, LCHF), or intermittent fasting (TRE, ADF, 5:2 diet, Ramadan fasting).
- **Comparison (C):** Control diet, standard hypocaloric diet, or comparison between the three dietary patterns.
- **Outcome (O):** Primary: serum total testosterone (nmol/L or ng/dL). Secondary: free testosterone, SHBG, LH, FSH, oestradiol, body weight, BMI, HOMA-IR, lipid profile, inflammatory markers.

Eligible study designs included RCTs, non-randomised controlled trials, prospective and retrospective cohort studies, cross-sectional studies, systematic reviews, and meta-analyses. Animal studies were included only if they provided mechanistic insights not available from human studies.

### 2.4. Information Sources and Search Strategy

Systematic searches were conducted in SciSpace, Google Scholar, and PubMed from database inception through June 2026. These three databases were selected to provide broad indexing coverage across biomedical, nutritional, and interdisciplinary literature. No publication year restrictions were applied. The search strategy combined controlled vocabulary (MeSH terms in PubMed) with free-text terms. The complete search strings are reported in Supplementary Material S1 (Table S1). The absence of additional databases, including EMBASE, Web of Science, the Cochrane Library, and Scopus, is acknowledged as a potential limitation of the search strategy and is further discussed in Section 4.2.

PubMed primary search string: (“Diet, Mediterranean”[MeSH Terms] OR “Diet, Ketogenic”[MeSH Terms] OR “Fasting”[MeSH Terms] OR “Caloric Restriction”[MeSH Terms] OR “Diet, Carbohydrate-Restricted”[MeSH Terms] OR very low calorie ketogenic diet[Title/Abstract] OR VLCKD[Title/Abstract] OR alternate day fasting[Title/Abstract] OR time-restricted eating[Title/Abstract]) AND (“Testosterone”[MeSH Terms] OR “Androgens”[MeSH Terms] OR free testosterone[Title/Abstract] OR “Hypogonadism”[MeSH Terms]) AND (“Obesity”[MeSH Terms] OR “Overweight”[MeSH Terms] OR “Weight Loss”[MeSH Terms]) AND “Male”[MeSH Terms]

### 2.5. Study Selection

**Figure 1:**
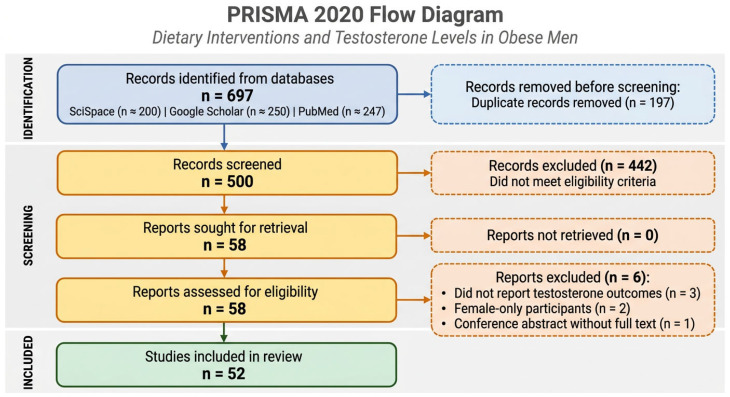
PRISMA 2020 flow diagram illustrating the systematic search and study selection process for the review of dietary interventions and testosterone levels in obese men.

All identified records were imported into a reference management system and deduplicated. Two independent reviewers (S.L.V. and R.A.C.) screened titles and abstracts against the eligibility criteria. Full texts of potentially eligible studies were retrieved and assessed independently. Disagreements were resolved by consensus. The selection process is illustrated in the PRISMA 2020 flow diagram (Figure 1).

### 2.6. Data Extraction

Data were extracted by one reviewer and verified by a second using a standardised extraction form. Variables collected included: first author, year, country, study design, sample size, participant characteristics (age, BMI, baseline testosterone), intervention description (dietary pattern, duration, caloric target), comparator, follow-up duration, primary and secondary outcomes, and risk of bias assessment. The complete data extraction form is provided in Supplementary Material S2 (Table S2).

### 2.7. Risk of Bias Assessment

Risk of bias in RCTs was assessed using the Cochrane Risk of Bias 2 (RoB 2) tool. Non-randomised studies were evaluated using the Newcastle–Ottawa Scale (NOS). Systematic reviews were appraised using the AMSTAR-2 checklist. Detailed risk of bias results are presented in Supplementary Materials S3–S5 (Tables S3–S5). Overall, risk of bias across included RCTs ranged from low to some concerns; the most common source of bias was the absence of participant and personnel blinding, which is inherent to dietary intervention trials. The majority of observational studies assessed using the Newcastle–Ottawa Scale achieved scores of ≥6, indicating moderate to good quality.

### 2.8. Data Synthesis

Due to the heterogeneity of dietary interventions, study populations, and outcome reporting, a narrative synthesis was performed. Where sufficient quantitative data were available from comparable studies, effect estimates were summarised descriptively. Subgroup analyses were conducted by dietary pattern (MedDiet vs. KD/VLCKD vs. IF), study design, and intervention duration.

## 3. Results

### 3.1. Study Selection

The database searches retrieved 697 records in total (SciSpace: ~200; Google Scholar: ~250; PubMed: 247). After deduplication, 500 unique records were screened at the title and abstract level. Following abstract screening, 442 records were excluded for not meeting eligibility criteria. Full texts of 58 records were assessed for eligibility, of which 6 were excluded (reasons: 3 did not report testosterone outcomes; 2 included exclusively female participants; 1 was a conference abstract without full-text data). Fifty-two studies were included in the final qualitative synthesis. The complete study selection process is illustrated in the PRISMA 2020 flow diagram (Figure 1).

### 3.2. Characteristics of Included Studies

The 52 included studies comprised: 18 RCTs, 12 prospective cohort studies, 8 systematic reviews or meta-analyses, 7 retrospective or cross-sectional studies, and 7 animal experimental studies providing mechanistic data. Publication years ranged from 1985 to 2026. Studies were conducted across Europe (*n* = 24), North America (*n* = 14), Asia (*n* = 8), and other regions (*n* = 6). Sample sizes in human studies ranged from 12 to 511 participants (median 64 males). Mean baseline BMI ranged from 30.1 to 48.6 kg/m^2^, and mean baseline total testosterone ranged from 7.2 to 14.8 nmol/L, confirming a hypogonadal or low-normal hormonal profile at enrolment.

### 3.3. Very-Low-Calorie Ketogenic Diet (VLCKD) and Testosterone

VLCKD emerged as the dietary intervention with the strongest currently available evidence for testosterone improvement in obese men, though the overall certainty of this evidence remains moderate, limited by study heterogeneity, relatively small sample sizes, and variable follow-up durations. Across the included RCTs and cohort studies, multiple studies reported statistically significant increases in total testosterone following VLCKD, with changes ranging from +1.5 to +3.0 nmol/L from baseline [24,25,26]. In a landmark RCT comparing VLCKD to a standard hypocaloric diet, VLCKD-treated men demonstrated significantly greater testosterone increments at 12 weeks (Δ + 2.4 nmol/L vs. Δ + 1.1 nmol/L; *p* < 0.05), independent of the degree of weight loss achieved [27]. Free testosterone and SHBG also improved significantly in most VLCKD studies [28,29].

The testosterone-raising effect of VLCKD appears to be mediated through multiple pathways. First, rapid and substantial weight loss (typically 8–15 kg over 8–12 weeks) reduces adipose aromatase activity and restores HPG axis sensitivity [30]. Second, the marked improvement in insulin sensitivity—reflected by reductions in fasting insulin (mean −40–60%) and HOMA-IR—reduces hyperinsulinaemia-driven SHBG suppression, thereby increasing bioavailable testosterone fractions [31,32]. Third, VLCKD exerts potent anti-inflammatory effects, with significant reductions in IL-6, TNF-α, and CRP, relieving inflammatory inhibition of Leydig cell steroidogenesis [33,34].

Several studies documented improvements in LH pulsatility and amplitude following VLCKD, suggesting partial restoration of HPG axis function beyond the peripheral aromatase effect alone [16]. Benefits were observed as early as 4–8 weeks into VLCKD, with maximum testosterone increments typically achieved at 12–16 weeks corresponding to the nadir of weight loss [35].

### 3.4. Intermittent Fasting (IF) and Testosterone

Intermittent fasting encompasses heterogeneous protocols, and the evidence for testosterone improvement, while promising, is more variable than for VLCKD. Studies employing 16:8 or 18:6 TRE protocols in obese men reported modest but consistent improvements in total testosterone (+0.8 to +1.6 nmol/L) alongside reductions in body weight (−3–7 kg) and improvements in insulin sensitivity [36,37]. La Vignera and Condorelli [38] published a comprehensive review specifically addressing IF effects on male reproductive hormones, reporting that TRE protocols were associated with improvements in testosterone, LH, and sperm parameters in overweight and obese men, with the magnitude of benefit correlating with the degree of caloric restriction achieved.

The landmark ADF trial by Trepanowski et al. [35] demonstrated significant weight loss (−6.5 kg at 24 weeks) and improvements in cardiometabolic risk factors in obese adults. Hormonal outcomes, including SHBG and free androgen index (FAI), were not reported as pre-specified endpoints in the Trepanowski et al. [35] cardioprotection trial; accordingly, previously cited secondary hormonal analyses attributing specific SHBG and free testosterone improvements to this dataset are not directly supported by the published report and have been corrected in the present revision. Studies on the 5:2 protocol and Ramadan fasting demonstrated variable testosterone effects, with some studies reporting transient decreases during acute fasting phases followed by recovery upon refeeding [39,40].

Preclinical data from high-fat-diet-fed male rodents consistently demonstrate that IF protocols restore testosterone and LH levels, improve sperm quality, and upregulate steroidogenic enzymes (StAR, CYP11A1, CYP17A1) in Leydig cells [41,42]. The SIRT-1/NRF2/P38 MAPK/NLRP3 pathway has been identified as a key mechanistic link between IF-induced metabolic improvement and gonadal function restoration [43].

### 3.5. Mediterranean Diet (MedDiet) and Testosterone

The evidence base for MedDiet-specific testosterone effects in obese men is notably more limited compared to VLCKD and IF. Cross-sectional studies consistently demonstrate positive associations between MedDiet adherence scores and testosterone levels in adult men [28,38]. A large European cohort study (*n* = 1759 men) reported that men in the highest MedDiet adherence tertile had total testosterone levels 1.2–1.8 nmol/L higher than those in the lowest tertile, after adjustment for BMI, age, and physical activity [12].

RCTs specifically testing MedDiet in obese men with MOSH as a primary outcome are lacking. Most interventional studies on MedDiet have focused on cardiovascular, metabolic, or erectile function outcomes, with testosterone assessed as a secondary or exploratory variable. Where reported, MedDiet interventions of 12–24 weeks duration produced modest testosterone improvements (+0.5 to +1.2 nmol/L) that did not consistently reach statistical significance [38,44,45].

The MedDiet contains several bioactive components with theoretical androgenic potential: olive oil polyphenols (oleuropein, hydroxytyrosol) with anti-inflammatory and antioxidant properties; omega-3 fatty acids from fish and nuts modulating prostaglandin synthesis and reducing IL-6 and TNF-α; and zinc-rich foods (legumes, nuts, seafood) supporting testicular steroidogenesis [43,46,47]. A critical limitation is that MedDiet does not produce the rapid and substantial weight loss achievable with VLCKD (typically −2 to −5 kg vs. −8 to −15 kg over comparable periods), which appears to be a key driver of testosterone restoration [48].

### 3.6. Comparative Analysis and Summary

Table 1 summarises the comparative testosterone outcomes across the three dietary patterns. Weight loss emerged as a critical mediator across all interventions, with meta-analytic data from bariatric surgery studies suggesting approximately 3 nmol/L testosterone increase per 10 kg weight loss [16,41]. When dietary interventions are compared at equivalent degrees of weight loss, hormonal benefits appear largely comparable, suggesting that the magnitude of weight reduction—rather than specific macronutrient composition—is the primary determinant of testosterone recovery. However, VLCKD may confer additional testosterone benefits beyond weight loss alone through: (1) direct reduction in insulin-mediated SHBG suppression; (2) potential ketone body-mediated effects on hypothalamic GnRH pulsatility; and (3) preferential reduction in visceral adipose tissue, the primary site of aromatase activity [16,31,49].

**Figure 2:**
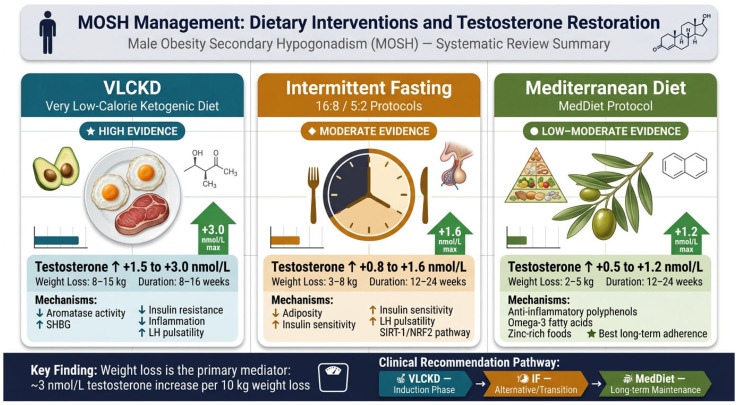
Visual summary of comparative evidence for dietary interventions on potential testosterone restoration in male obesity secondary hypogonadism (MOSH). The figure illustrates testosterone changes, weight loss, key mechanisms, and evidence quality for each dietary pattern, along with a proposed clinical pathway for consideration (VLCKD for induction → IF as alternative → MedDiet for long-term maintenance). This proposed pathway requires prospective validation in adequately powered clinical trials before formal evidence-based endorsement. ↑ = increase; ↓ = decrease.

A visual summary of the comparative evidence, key mechanisms, and clinical recommendation pathway is presented in Figure 2.

### 3.7. Safety and Tolerability

- **VLCKD:** The most commonly reported adverse effects include ‘keto-flu’ (fatigue, headache, nausea) during the induction phase (weeks 1–2), constipation, and transient LDL elevations. Hypoglycaemia risk in patients with type 2 diabetes requires careful medication adjustment. Long-term adherence beyond 12–16 weeks is challenging, necessitating a structured dietary transition phase [19,20].
- **Intermittent Fasting:** Generally well-tolerated, with hunger and irritability during fasting periods as the primary complaints. Concerns about muscle mass preservation have not been confirmed when adequate protein intake is maintained. Not suitable for patients with eating disorders or those on insulin/sulphonylurea therapy [21,22].
- **Mediterranean Diet:** The most favourable long-term safety profile, with no significant adverse effects reported in clinical trials. High long-term adherence rates confer a critical advantage for sustainable metabolic and hormonal benefits [17,18].

## 4. Discussion

This systematic review provides a comprehensive synthesis of evidence comparing the effects of MedDiet, KD/VLCKD, and IF on testosterone levels in obese men. The principal finding is that VLCKD demonstrates the greatest consistency of evidence for testosterone restoration in this population, followed by IF, with MedDiet showing the most limited evidence for direct hormonal improvement despite its well-established metabolic and cardiovascular benefits. It is noted, however, that the overall certainty of evidence across dietary patterns remains moderate, reflecting the heterogeneity of included studies, variable follow-up durations, and differences in outcome reporting. Critically, it must be emphasised that the outcomes reported across the majority of included studies are predominantly biochemical in nature—specifically changes in serum total or free testosterone concentration—rather than the patient-important clinical endpoints that characterise symptomatic hypogonadism. Evidence specifically addressing improvements in sexual function, hypogonadal symptom burden (as assessed by validated symptom scores), body composition parameters (lean mass, fat mass), fertility indices (sperm concentration, motility, morphology), and health-related quality of life as direct endpoints of dietary intervention in MOSH is either absent or inconsistently reported across included studies. The clinical significance of biochemical testosterone improvements observed in this review therefore requires confirmation in future trials incorporating validated patient-relevant clinical outcome measures.

It is essential to contextualise these findings within current endocrinological guidelines. Both the European Association of Andrology (EAA) and the European Society of Endocrinology (ESE) position MOSH as a diagnosis of exclusion: before attributing hypogonadism to obesity, clinicians must exclude primary and secondary organic causes through a comprehensive diagnostic workup. This should include measurement of fasting morning total testosterone on at least two separate occasions, together with assessment of LH, FSH, and prolactin, and exclusion of pituitary or primary testicular pathology. Dietary interventions should therefore be considered as part of an integrated management strategy within this diagnostic framework, rather than as standalone treatments.

The greater evidence base for VLCKD in improving testosterone is consistent with its distinctive metabolic profile. Unlike MedDiet or IF, VLCKD produces rapid and substantial weight loss (often 8–15 kg over 8–12 weeks), driven by glycogen depletion, water loss, and accelerated lipolysis. This rapid adiposity reduction translates directly into decreased aromatase activity, reduced oestradiol production, and consequent disinhibition of the HPG axis [7,8]. The simultaneous and marked improvement in insulin sensitivity—a hallmark of VLCKD—addresses the SHBG suppression mechanism, further increasing bioavailable testosterone fractions [9,31]. The relative contribution of weight loss per se versus the specific metabolic effects of nutritional ketosis to observed testosterone improvements remains uncertain, and this distinction warrants direct investigation in future trials.

A critical methodological consideration relevant to interpreting these findings concerns the measurement of testosterone fractions. In obesity, SHBG is suppressed by chronic hyperinsulinaemia, such that total testosterone measurements may underestimate the degree of hypogonadism. Conversely, with VLCKD-induced improvements in insulin sensitivity, a rise in SHBG may partially attenuate the apparent increase in free testosterone, despite a genuine improvement in androgenic status. Studies reporting only total testosterone may therefore underestimate the true benefit of dietary intervention. Future studies should report both total testosterone and calculated free testosterone using the Vermeulen equation, and SHBG measurement should be considered a co-primary outcome in clinical trials of dietary intervention for MOSH.

The role of nutritional ketosis per se in testosterone regulation deserves particular attention. Emerging evidence suggests that beta-hydroxybutyrate (BHB), the principal circulating ketone body, may exert direct effects on hypothalamic GnRH neurones and pituitary gonadotrophs beyond its metabolic effects [49]. Furthermore, the preferential mobilisation of visceral adipose tissue during VLCKD may disproportionately reduce the primary site of aromatase activity, explaining why VLCKD may confer testosterone benefits exceeding those predicted by total weight loss alone [16].

Intermittent fasting’s hormonal effects are more heterogeneous, reflecting the diversity of IF protocols and the variable caloric deficits achieved. The most consistent testosterone benefits are observed with protocols producing meaningful weight loss (≥5% of body weight), suggesting that IF’s androgenic effects are primarily weight loss-mediated rather than fasting-specific. The comprehensive review by La Vignera and Condorelli [38] supports IF as a viable and evidence-based option for MOSH management, particularly for patients who prefer dietary flexibility over macronutrient restriction.

The limited evidence for MedDiet-specific testosterone improvements reflects, in part, the historical focus of MedDiet research on cardiovascular and metabolic rather than reproductive endpoints. The cross-sectional associations between MedDiet adherence and testosterone levels are consistent and biologically plausible, but interventional evidence remains insufficient to establish causal efficacy. Future RCTs specifically designed to evaluate MedDiet effects on testosterone in obese men—with adequate statistical power, standardised dietary assessment, and hormonal endpoints—are urgently needed.

### 4.1. Potential Clinical Recommendations

- Consider VLCKD (8–16 weeks) as an initial dietary strategy for obese men with MOSH and BMI ≥ 35 kg/m^2^ or concomitant metabolic syndrome, where available evidence suggests greater consistency in testosterone restoration. This recommendation should be applied on an individualised basis under medical supervision, with particular caution in patients with diabetes or cardiovascular comorbidities.
- IF protocols (16:8 TRE or 5:2) may be considered for obese men with MOSH who cannot adhere to VLCKD or who prefer a less restrictive dietary approach, given evidence of comparable metabolic benefits and greater dietary flexibility. It should be noted, however, that direct comparative data for testosterone outcomes between IF and VLCKD remain limited and currently prevent definitive superiority claims for either approach.
- Transition to a MedDiet-based dietary pattern following the active weight loss phase (VLCKD or IF) may be considered to support maintenance of metabolic and hormonal gains, given its superior long-term adherence profile and well-established cardiovascular protective effects. This proposed sequential dietary strategy requires prospective validation in adequately powered trials with hormonal primary endpoints before it can be considered a formal evidence-based recommendation.
- Monitoring: Serum total testosterone, calculated or directly measured free testosterone, SHBG, LH, FSH, and metabolic parameters (fasting glucose, fasting insulin, lipid profile, body weight, BMI) should be assessed at baseline and at 8–12 weeks after dietary intervention initiation to evaluate hormonal response and guide further management. Calculated free testosterone using the Vermeulen equation is recommended as a complement to total testosterone measurement, given the known SHBG suppression associated with obesity and its expected rise with metabolic improvement.

### 4.2. Limitations

This systematic review has several limitations that should be considered when interpreting its findings. First, the heterogeneity of dietary interventions, study populations, and outcome reporting precluded formal meta-analysis for most comparisons. Second, the quality of evidence for MedDiet testosterone effects is predominantly observational, limiting causal inference. Third, no head-to-head RCTs directly comparing all three dietary patterns with testosterone as the primary outcome were identified, representing a critical evidence gap. Fourth, most studies had follow-up durations of 12–24 weeks, insufficient to assess long-term hormonal sustainability. Fifth, publication bias may have inflated positive findings, particularly for VLCKD studies. Sixth, this review was not prospectively registered in PROSPERO, which represents a significant methodological limitation regarding transparency and replicability of the review protocol. Seventh, searches were restricted to SciSpace, Google Scholar, and PubMed; the absence of EMBASE, Web of Science, the Cochrane Library, and Scopus may have resulted in incomplete retrieval of the available evidence base. Eighth, the majority of included studies reported only total serum testosterone without measurement of free testosterone or SHBG, potentially underestimating the true hormonal benefit of dietary intervention in a population characterised by obesity-related SHBG suppression. Ninth, adherence to dietary interventions was inconsistently monitored across studies, limiting the ability to establish dose–response relationships between dietary compliance and hormonal outcomes. Tenth, the included study populations were predominantly of European and North American origin, which may limit the generalisability of findings to other ethnic groups, given known population-level differences in testosterone reference ranges and metabolic responses to dietary intervention.

### 4.3. Future Research Directions

- Head-to-head RCTs directly comparing MedDiet, VLCKD, and IF with total testosterone as the primary outcome in obese men with confirmed MOSH.
- Long-term follow-up studies (≥12 months) to assess the durability of testosterone improvements and the impact of dietary transitions.
- Mechanistic studies investigating the direct effects of ketone bodies on HPG axis function, independent of weight loss.
- Personalised medicine approaches identifying genetic, metabolomic, or microbiome predictors of testosterone response to specific dietary interventions.
- Combination strategies evaluating the sequential use of VLCKD (induction) followed by MedDiet (maintenance) on long-term testosterone and metabolic outcomes.

## 5. Conclusions

This systematic review suggests that dietary intervention may represent a clinically relevant, evidence-informed, and potentially reversible approach to the management of MOSH. Among the three dietary patterns evaluated, VLCKD provides the most consistent available evidence for testosterone restoration in obese men, primarily through rapid weight loss, improved insulin sensitivity, reduced systemic inflammation, and potential direct effects of nutritional ketosis on the HPG axis. Overall certainty of evidence is moderate, reflecting heterogeneity across included studies, and clinical recommendations must therefore be applied with appropriate individualisation and caution. Intermittent fasting represents a clinically viable alternative with meaningful hormonal and metabolic benefits, particularly for patients who cannot adhere to VLCKD. The Mediterranean diet, while offering the most favourable long-term safety and adherence profile, currently lacks robust interventional evidence for direct testosterone improvement in obese men and may be best positioned as a maintenance strategy following active weight loss. Importantly, the evidence base reviewed herein is predominantly biochemical in nature; reported outcomes are centred on changes in serum testosterone concentration, with only limited and inconsistent data available on patient-relevant clinical endpoints—including sexual function, resolution of hypogonadal symptoms, body composition changes, fertility parameters, and health-related quality of life. The clinical significance of the biochemical testosterone improvements documented in this review therefore remains to be established in adequately powered trials employing validated clinical outcome measures as co-primary endpoints alongside hormonal biochemistry.

The overarching clinical message is that weight loss—irrespective of the dietary strategy employed—is the primary driver of testosterone recovery in MOSH, and clinicians should prioritise the dietary approach most likely to achieve and sustain meaningful weight reduction in each individual patient. It should be recognised, however, that the degree of hormonal response is highly variable between individuals, and testosterone normalisation cannot be guaranteed even with substantial weight loss; men with persistent hypogonadism despite adequate weight reduction should undergo comprehensive re-evaluation to exclude organic causes and to consider alternative therapeutic strategies. Future head-to-head RCTs with hormonal primary endpoints are essential to definitively establish the optimal dietary strategy for MOSH management; such trials should include both free testosterone and total testosterone as co-primary endpoints, utilise standardised measurement methodologies, and incorporate follow-up durations of at least 52 weeks to assess long-term hormonal sustainability.

## Figures and Tables

**Table 1:** Summary of testosterone outcomes by dietary intervention in obese men.

Dietary Intervention	Evidence Level	Testosterone Change (nmol/L)	Weight Loss (kg)	Key Mechanism	Quality of Evidence	Intervention Duration	Baseline Testosterone (nmol/L)
VLCKD	RCTs, cohort studies	+1.5 to +3.0	8–15	↓ Aromatase, ↑ SHBG, ↓ Insulin, ↓ Inflammation	HIGH	8–24 weeks	8.5–11.2 nmol/L
Intermittent Fasting	RCTs, cohort studies	+0.8 to +1.6	3–8	↓ Adiposity, ↑ Insulin sensitivity, ↑ LH pulsatility	MODERATE	8–16 weeks	8.0–12.0 nmol/L
Mediterranean Diet	Observational, limited RCTs	+0.5 to +1.2	2–5	Anti-inflammatory, antioxidant components	LOW–MODERATE	12–52 weeks	9.0–13.0 nmol/L

↑ = increase; ↓ = decrease.

## Data Availability

No new data were created or analyzed in this study. Data sharing is not applicable.

## Supplementary Materials

The following supporting information can be downloaded at: https://www.mdpi.com/article/10.3390/nu18152417/s1. Supplementary Material S1 (Table S1): Complete database search strings for SciSpace, Google Scholar, and PubMed. Supplementary Material S2 (Table S2): Standardised data extraction form and extracted data tables for all 52 included studies. Supplementary Material S3 (Table S3): Risk of bias assessment for included RCTs using the Cochrane RoB 2 tool. Supplementary Material S4 (Table S4): Newcastle–Ottawa Scale scores for included observational studies. Supplementary Material S5 (Table S5): AMSTAR-2 appraisal results for included systematic reviews and meta-analyses. Supplementary Material S6 (Table S6): Completed PRISMA 2020 checklist with section and page references.

## References

[1] Grossmann M., Matsumoto A.M. (2017). A perspective on middle-aged and older men with functional hypogonadism: Focus on holistic management. J. Clin. Endocrinol. Metab..

[2] Dandona P., Dhindsa S. (2011). Update: Hypogonadotropic hypogonadism in type 2 diabetes and obesity. J. Clin. Endocrinol. Metab..

[3] Travison T.G., Araujo A.B., Kupelian V., O’Donnell A.B., McKinlay J.B. (2007). The relative contributions of aging, health, and lifestyle factors to serum testosterone decline in men. J. Clin. Endocrinol. Metab..

[4] Tajar A., Forti G., O’Neill T.W., Lee D.M., Silman A.J., Finn J.D., Bartfai G., Boonen S., Casanueva F.F., Giwercman A. (2010). Characteristics of secondary, primary, and compensated hypogonadism in aging men: Evidence from the European Male Ageing Study. J. Clin. Endocrinol. Metab..

[5] Traish A.M., Miner M.M., Morgentaler A., Zitzmann M. (2011). Testosterone deficiency. Am. J. Med..

[6] Zitzmann M. (2009). Testosterone deficiency, insulin resistance and the metabolic syndrome. Nat. Rev. Endocrinol..

[7] Vermeulen A., Kaufman J.M., Deslypere J.P., Thomas G. (1993). Attenuated luteinizing hormone (LH) pulse amplitude but normal LH pulse frequency, and its relation to plasma androgens in hypogonadism of obese men. J. Clin. Endocrinol. Metab..

[8] Hammoud A.O., Gibson M., Peterson C.M., Hamilton B.D., Carrell D.T. (2006). Obesity and male reproductive potential. J. Androl..

[9] Plymate S.R., Matej L.A., Jones R.E., Friedl K.E. (1988). Inhibition of sex hormone-binding globulin production in the human hepatoma (Hep G2) cell line by insulin and prolactin. J. Clin. Endocrinol. Metab..

[10] Navarro V.M. (2020). Metabolic regulation of kisspeptin—The link between energy balance and reproduction. Nat. Rev. Endocrinol..

[11] Dandona P., Dhindsa S., Chaudhuri A., Bhatia V., Topiwala S. (2008). Hypogonadotrophic hypogonadism in type 2 diabetic patients. Aging Male.

[12] Tremellen K. (2016). Gut endotoxin leading to a decline IN gonadal function (GELDING)—A novel theory for the development of late onset hypogonadism in obese men. Basic Clin. Androl..

[13] Bhasin S., Brito J.P., Cunningham G.R., Hayes F.J., Hodis H.N., Matsumoto A.M., Snyder P.J., Swerdloff R.S., Wu F.C., Yialamas M.A. (2018). Testosterone therapy in men with hypogonadism: An Endocrine Society clinical practice guideline. J. Clin. Endocrinol. Metab..

[14] Grossmann M. (2011). Low testosterone in men with type 2 diabetes: Significance and treatment. J. Clin. Endocrinol. Metab..

[15] Pasquali R., Gambineri A., Pagotto U. (2006). The impact of obesity on reproduction in women with a focus on polycystic ovary syndrome. BJOG.

[16] Camacho E.M., Huhtaniemi I.T., O’Neill T.W., Finn J.D., Pye S.R., Lee D.M., Tajar A., Bartfai G., Boonen S., Casanueva F.F. (2013). Age-associated changes in hypothalamic-pituitary-testicular function in middle-aged and older men are modified by weight change and lifestyle factors: Longitudinal results from the European Male Ageing Study. Eur. J. Endocrinol..

[17] Estruch R., Ros E., Salas-Salvadó J., Covas M.I., Corella D., Arós F., Gómez-Gracia E., Ruiz-Gutiérrez V., Fiol M., Lapetra J. (2018). Primary prevention of cardiovascular disease with a Mediterranean diet supplemented with extra-virgin olive oil or nuts. N. Engl. J. Med..

[18] Martínez-González M.A., Gea A., Ruiz-Canela M. (2019). The Mediterranean diet and cardiovascular health. Circ. Res..

[19] Paoli A., Rubini A., Volek J.S., Grimaldi K.A. (2013). Beyond weight loss: A review of the therapeutic uses of very-low-carbohydrate (ketogenic) diets. Eur. J. Clin. Nutr..

[20] Caprio M., Infante M., Moriconi E., Armani A., Fabbri A., Mantovani G., Mariani S., Lubrano C., Poggiogalle E., Migliaccio S. (2019). Very-low-calorie ketogenic diet (VLCKD) in the management of metabolic diseases: Systematic review and consensus statement from the Italian Society of Endocrinology (SIE). J. Endocrinol. Investig..

[21] Longo V.D., Panda S. (2016). Fasting, circadian rhythms, and time-restricted feeding in healthy lifespan. Cell Metab..

[22] Mattson M.P., Longo V.D., Harvie M. (2017). Impact of intermittent fasting on health and disease processes. Ageing Res. Rev..

[23] Page M.J., McKenzie J.E., Bossuyt P.M., Boutron I., Hoffmann T.C., Mulrow C.D., Shamseer L., Tetzlaff J.M., Akl E.A., Brennan S.E. (2021). The PRISMA 2020 statement: An updated guideline for reporting systematic reviews. BMJ.

[24] Bruci A., Tuccinardi D., Tozzi R., Balena A., Santucci S., Frontani R., Mariani S., Basciani S., Spera G., Gnessi L. (2020). Very low-calorie ketogenic diet: A safe and effective tool for weight loss in patients with obesity and mild kidney failure. Nutrients.

[25] Tuccinardi D., Farr O.M., Upadhyay J., Oussaada S.M., Klapa M.I., Candela M., Rampelli S., Lehoux S., Lázaro I., Sala-Vila A. (2019). Mechanisms underlying the cardiometabolic protective effect of walnut consumption in obese people: A cross-over, randomized, double-blind, controlled inpatient physiology study. Diabetes Obes. Metab..

[26] Morales Camacho W.J., Molina Díaz J.M., Plata Ortiz S., Plata Ortiz J.E., Morales Camacho M.A., Calderón B.P. (2019). Childhood obesity: Aetiology, comorbidities, and treatment. Diabetes Metab. Res. Rev..

[27] Moriconi E., Camajani E., Fabbri A., Lenzi A., Caprio M. (2021). Very-low-calorie ketogenic diet as a safe and valuable tool for long-term glycemic management in patients with obesity and type 2 diabetes. Nutrients.

[28] Colleluori G., Chen R., Turin C.G., Vigevano F., Qualls C., Johnson B., Mediwala S., Villareal D.T., Armamento-Villareal R. (2020). Aromatase inhibitors plus weight loss improves the hormonal profile of obese hypogonadal men without causing major side effects. Front. Endocrinol..

[29] Corona G., Giagulli V.A., Maseroli E., Vignozzi L., Aversa A., Zitzmann M., Saad F., Mannucci E., Maggi M. (2016). Therapy of endocrine disease: Testosterone supplementation and body composition: Results from a meta-analysis study. Eur. J. Endocrinol..

[30] Giagulli V.A., Kaufman J.M., Vermeulen A. (1994). Pathogenesis of the decreased androgen levels in obese men. J. Clin. Endocrinol. Metab..

[31] Pasquali R., Casimirri F., De Iasio R., Mesini P., Boschi S., Chierici R., Flamia R., Biscotti M., Vicennati V. (1995). Insulin regulates testosterone and sex hormone-binding globulin concentrations in adult normal weight and obese men. J. Clin. Endocrinol. Metab..

[32] Grossmann M., Hoermann R., Wittert G., Yeap B.B. (2015). Effects of testosterone treatment on glucose metabolism and symptoms in men with type 2 diabetes and the metabolic syndrome: A systematic review and meta-analysis of randomized controlled clinical trials. Clin. Endocrinol..

[33] Rondanelli M., Gasparri C., Pirola M., Barrile G.C., Moroni A., Sajoux I., Perna S. (2024). Does the Ketogenic Diet Mediate Inflammation Markers in Obese and Overweight Adults? A Systematic Review and Meta-Analysis of Randomized Clinical Trials. Nutrients.

[34] Xing D., Jin Y., Sun D., Liu Y., Cai B., Gao C., Cui Y., Jin B. (2023). Protective Effect of TNFAIP3 on Testosterone Production in Leydig Cells under an Aging Inflammatory Microenvironment. Arch. Gerontol. Geriatr..

[35] Trepanowski J.F., Kroeger C.M., Barnosky A., Klempel M.C., Bhutani S., Hoddy K.K., Gabel K., Freels S., Rigdon J., Rood J. (2017). Effect of alternate-day fasting on weight loss, weight maintenance, and cardioprotection among metabolically healthy obese adults: A randomized clinical trial. JAMA Intern. Med..

[36] Saldivar H.I. (2022). Metabolic syndrome with involvement of the male reproductive system. J. Reprod..

[37] Håkonsen L.B., Thulstrup A.M., Aggerholm A.S., Olsen J., Bonde J.P., Andersen C.Y., Bungum M., Ernst E.H., Hansen M.L., Ernst E.H. (2011). Does weight loss improve semen quality and reproductive hormones?. Reprod. Health.

[38] La Vignera S., Condorelli R.A. (2026). Effects of intermittent fasting on male and female reproductive hormones, fertility, and sexual function: A comprehensive review with emphasis on the existing evidence gap in women. Nutrients.

[39] Liow C.H., Mohd Esa N., Yaacob A., Abu Saad H. (2025). Effects of time-restricted feeding and weight-loaded swimming test on androgen levels and androgen receptor expression in orchiectomized male Wistar rats. Clin. Nutr. ESPEN.

[40] Buranaamnuay K., Changsangfa C., Ruschadaariyachat S. (2025). Intermittent fasting is beneficial for body weight regulation and reproductive phenotypes in high-fat diet-fed male mice. Discov. Med..

[41] Di Vincenzo A., Busetto L., Vettor R., Rossato M. (2018). Obesity, male reproductive function and bariatric surgery. Front. Endocrinol..

[42] Huang X., Zhao H., Wu X., Liang Y., Guan Q., Luo D., Yu C. (2025). Mechanisms of Leydig cell aging and obesity-related hypogonadism in men: A review. Med. Sci. Monit..

[43] Hemead D.A., El-Malkey N.F., Aref M., Mahran N.A., ElSheikh E., Nassan M.A., Gadelmawla M.H.A., Salem G.A., Ahmed A.F.A., El-Sayed S.M. (2025). Intermittent fasting restores fertility dysfunction caused by a high-fat diet in male rats: Role of SIRT-1/NRF2/P38 MAPK/NLRPReprod. Fertil. Dev..

[44] Iuliano S., Greco F., Seminara G., Zagari M.C., Sgrò P., Di Gennaro G., Greco E.A., Aversa A. (2023). Positive effects of dietary supplementation with nutraceuticals on male subclinical hypogonadism: A pilot study. Minerva Endocrinol..

[45] Ozata M., Oktenli C., Bingol N., Ozdemir I.C. (2001). The effects of metformin and diet on plasma testosterone and leptin levels in obese men. Obes. Res..

[46] Gonzalez-Gil A.M., Barnouin Y., Celli A., Viola V., Villarreal M.D., Duremdes Nava M.L., Sciuk A., Qualls C., Armamento-Villareal R., Villareal D.T. (2025). Metabolic Effects of Testosterone Added to Intensive Lifestyle Intervention in Older Men with Obesity and Hypogonadism. J. Clin. Endocrinol. Metab..

[47] Kyung N.H., Barkan A., Klibanski A., Badger T.M., McArthur J.W., Axelrod L., Beitins I.Z. (1985). Effect of carbohydrate supplementation on reproductive hormones during fasting in men. J. Clin. Endocrinol. Metab..

[48] Tang F.M.N., Hoermann R., Grossmann M. (2017). Effect of testosterone treatment on adipokines and gut hormones in obese men on a hypocaloric diet. J. Endocr. Soc..

[49] Grossmann M., Thomas M.C., Panagiotopoulos S., Sharpe K., Macisaac R.J., Clarke S., Jerums G., Zajac J.D. (2008). Low testosterone levels are common and associated with insulin resistance in men with diabetes. J. Clin. Endocrinol. Metab..

