# Supplementary material for: Dietary Interventions and Testosterone Levels in Obese Men: A Systematic Review Comparing the Mediterranean Diet, Ketogenic Diet, and Intermittent Fasting
Source: Nutrients. 2026 Jul 24;18(15):2417. doi: 10.3390/nu18152417 (PMC13467967; doi:10.3390/nu18152417)
Supplement: Supplementary file 1 [file nutrients-18-02417-s001.zip › nutrients-4434142-supplementary.pdf]

Supplementary Materials

Dietary Interventions and Testosterone Levels in Obese Men: A Systematic Review Comparing the Mediterranean Diet, Ketogenic Diet, and Intermittent Fasting

La Vignera S. * and Condorelli R.A.

Department of Clinical and Experimental Medicine, University of Catania, Via S. Sofia 78, 95123 Catania, Italy

* Correspondence: sandrolavignera@unict.it

Table S1. Complete database search strings for SciSpace, Google Scholar, and PubMed

Database: PubMed

Search performed: June 2026

```
(Mediterranean diet[Title/Abstract] OR ketogenic diet[Title/Abstract] OR
intermittent fasting[Title/Abstract] OR caloric restriction[Title/Abstract] OR
low-carbohydrate diet[Title/Abstract] OR VLCKD[Title/Abstract] OR
very low calorie ketogenic diet[Title/Abstract] OR time-restricted
eating[Title/Abstract] OR
alternate day fasting[Title/Abstract])
AND
(testosterone[Title/Abstract] OR androgens[Title/Abstract] OR
hypogonadism[Title/Abstract] OR
androgen deficiency[Title/Abstract] OR SHBG[Title/Abstract] OR luteinizing
hormone[Title/Abstract])
AND
(obesity[Title/Abstract] OR obese[Title/Abstract] OR weight loss[Title/Abstract]
OR
overweight[Title/Abstract] OR adiposity[Title/Abstract] OR BMI[Title/Abstract])
AND
male[Title/Abstract]
```

Results: ~247 records

Database: Google Scholar

Search performed: June 2026

Search string 1:

```
"dietary intervention" "testosterone" "obese men" systematic review
```

Search string 2:

```
"ketogenic diet" OR "VLCKD" "testosterone" "obesity" "hypogonadism"
```

Search string 3:

```
"intermittent fasting" "testosterone" "male" "obesity" clinical trial
```

Search string 4:

```
"Mediterranean diet" "testosterone" "men" "weight loss"
```

Results: ~250 records

Database: SciSpace

Search performed: June 2026

Search string:

```
dietary interventions testosterone obese men systematic review
Mediterranean ketogenic intermittent fasting hypogonadism MOSH
```

Results: ~200 records

Total records identified: 697 Duplicates removed: 197 Records screened: 500

Table S2. Standardised data extraction form and extracted data tables for all 52 included studies

The following variables were extracted for each included study:

Variable	Description
First author	Last name and initials
Year	Year of publication
Country	Country of study conduct
Study design	RCT, cohort, cross-sectional, animal, systematic review
Sample size	Total N; N males
Age (years)	Mean ± SD or range
BMI (kg/m^2)	Mean ± SD at baseline
Baseline testosterone	Total testosterone nmol/L (mean ± SD)
Dietary intervention	Type, description, duration, caloric target

Comparator	Control diet / standard hypocaloric / other intervention
Follow-up duration	Weeks
Primary outcome	Total testosterone change (nmol/L or ng/dL)
Secondary outcomes	Free testosterone, SHBG, LH, FSH, E2, body weight, BMI, HOMA-IR, lipid profile, inflammatory markers
Statistical method	Test used for primary outcome
Key result	Direction and magnitude of testosterone change
Risk of bias	Tool used and overall judgement
Notes	Any relevant comments

Table S3. Risk of bias assessment for included RCTs using the Cochrane RoB 2 tool

Study	D1: Randomisation	D2: Deviations	D3: Missing Data	D4: Outcome Measurement	D5: Selective Reporting	Overall
Moriconi et al. 2021 [27]	Low	Low	Low	Low	Low	**Low**
Trepanowski et al. 2017 [35]	Low	Low	Some concerns	Low	Low	**Some concerns**
Bruci et al. 2020 [24]	Low	Some concerns	Low	Low	Low	**Some concerns**
Tuccinardi et al. 2019 [25]	Low	Low	Low	Low	Low	**Low**
Ozata et al. 2001 [45]	Some concerns	Some concerns	Low	Low	Low	**Some concerns**
Tang et al. 2017 [48]	Low	Low	Some concerns	Low	Low	**Some concerns**

D1: Bias arising from the randomisation process; D2: Bias due to deviations from intended interventions; D3: Bias due to missing outcome data; D4: Bias in measurement of the outcome; D5: Bias in selection of the reported result.

Table S4. Newcastle–Ottawa Scale scores for included observational studies

Study	Design	Selection (max 4)	Comparability (max 2)	Outcome/Exposure (max 3)	Total Score (max 9)	Quality
Camacho et al. 2013 [16]	Cohort	4	2	3	**9**	High
Giagulli et al. 1994 [30]	Cross-sectional	3	2	2	**7**	Moderate–High
Saldivar et al. 2022 [36]	Cross-sectional	3	1	2	**6**	Moderate
Tremellen et al. 2016 [12]	Cross-sectional	3	2	2	**7**	Moderate–High
Pasquali et al. 1995 [31]	Cohort	4	2	3	**9**	High
Corona et al. 2016 [29]	Cohort	3	2	3	**8**	High

Scores ≥ 7 considered high quality; 5–6 moderate quality; < 5 low quality.

Table S5. AMSTAR-2 appraisal results for included systematic reviews and meta-analyses

Study	Protocol Registered	Comprehensive Search	Duplicate Screening	Full-Text Exclusion Reasons	Risk of Bias Assessed	Appropriate Meta-analysis	Publication Bias	Overall Rating
La Vignera & Condorelli 2026 [38]	No	Yes	Yes	Yes	Yes	N/A	Not assessed	**Moderate**
Di Vincenzo et al. 2018 [41]	No	Partial	Yes	Yes	Yes	N/A	Not assessed	**Low–Moderate**

Overall rating based on AMSTAR-2 critical and non-critical domain assessment.

Table S6. Completed PRISMA 2020 checklist with section and page references

Section / Topic	Item #	Checklist Item	Location in Manuscript
**TITLE**			
Title	1	Identify the report as a systematic review.	Title page
**ABSTRACT**			
Abstract	2	Provide a structured summary including background, objectives, methods, results, conclusions, and registration.	Abstract
**INTRODUCTION**			
Rationale	3	Describe the rationale for the review in the context of existing knowledge.	Section 1
Objectives	4	Provide an explicit statement of the objective(s) or question(s) the review addresses.	Section 1 (final paragraph)
**METHODS**			
Protocol and registration	5	Indicate whether a review protocol exists and where it can be accessed; provide registration information.	Section 2.2
Eligibility criteria	6	Specify eligibility criteria for the review.	Section 2.3
Information sources	7	Describe all information sources used in the search and the date last searched.	Section 2.4
Search strategy	8	Present the full search strategy for at least one database, including filters applied.	Section 2.4 + Table S1
Selection process	9	Specify the methods used to decide whether a study met the inclusion criteria.	Section 2.5
Data collection process	10	Describe the methods used to collect data from reports.	Section 2.6 + Table S2
Data items	11	List and define all outcomes for which data were sought.	Section 2.6
Study risk of bias	12	Specify the methods used to assess risk of bias in the included studies.	Section 2.7
Effect measures	13	Specify for each outcome the effect measure(s) used in the synthesis.	Section 2.8
Synthesis methods	14	Describe the methods used to synthesise results.	Section 2.8

Reporting bias assessment	15	Describe methods used to assess risk of bias due to missing results.	Section 4.2
Certainty assessment	16	Describe the methods used to assess certainty of the body of evidence.	Section 2.7
**RESULTS**			
Study selection	17	Describe the results of the search and selection process, with a flow diagram.	Section 3.1, Figure 1
Study characteristics	18	Cite each included study and present its characteristics.	Section 3.2
Risk of bias in studies	19	Present assessments of risk of bias for each included study.	Section 3.7, Tables S3–S5
Results of individual studies	20	Present results for all outcomes for each included study.	Sections 3.3–3.6
Results of syntheses	21	Summarise the results of each synthesis.	Sections 3.3–3.6, Table 1
Reporting biases	22	Present assessments of risk of bias due to missing results.	Section 4.2
Certainty of evidence	23	Present assessments of certainty of evidence.	Table 1, Section 3.6
**DISCUSSION**			
Discussion	24a	Provide a general interpretation of the results in the context of other evidence.	Section 4
Limitations	24b	Discuss any limitations of the evidence included in the review.	Section 4.2
Conclusions	24c	Provide a general interpretation and implications for practice and research.	Section 5
**OTHER INFORMATION**			
Registration and protocol	25	Provide registration information for the review.	Section 2.2
Support	26	Describe sources of financial or non-financial support.	Funding
Competing interests	27	Declare any competing interests of review authors.	Conflicts of Interest

This systematic review was conducted and reported in full accordance with the PRISMA 2020 statement (Page et al., BMJ 2021;372:n71). All 27 checklist items have been addressed where applicable.
